# Nurturing Bonds: A Qualitative Exploration of Breastfeeding and Responsive Feeding Practices in Samoan Mother-Infants Dyads

**DOI:** 10.1002/ajhb.70241

**Published:** 2026-04

**Authors:** Bertacchi Victoria, Daiy Katherine, Lowry Kemper, Vesi Lupesina, Filipo Vaimoana, Faaselele-Savusa Kima, Pérez-Escamilla Rafael, Hromi-Fiedler Amber, Naseri Take, Soti-Ulberg Christina, Abraham Jyothi, Richard G. Bribiescas, Nicola L. Hawley

**Affiliations:** 1 Department of Anthropology, Yale University, New Haven, Connecticut, USA; 2 Department of Anthropology, The Ohio State University, Columbus, Ohio, USA; 3 Obesity, Lifestyle and Genetic Adaptations (OLaGA) Study Group, Apia, Samoa; 4 Yale School of Public Health, Yale University, New Haven, Connecticut, USA; 5 Ministry of Health, Apia, Samoa; 6 School of Nursing, National University of Samoa, Apia, Samoa

**Keywords:** breastfeeding, maternal-infant health, qualitative exploration, responsive feeding, Samoa

## Abstract

**Introduction:**

Little is known about whether perceptions in Samoa of human milk composition and quality play a role in the decline in exclusive breastfeeding with age, the introduction of solid foods, or the degree to which mothers are utilizing responsive feeding practices. To explore this topic, we qualitatively explored feeding practices in Samoan families to identify areas in which the introduction of responsive feeding education may support and supplement the current breastfeeding promotion success.

**Methods:**

In 2020–2021 we conducted one-on-one structured interviews with *n* = 100 mothers of infants aged 2–4 months. A subset of the sample (*n* = 25) was asked to take part in focus groups approximately a year later to gain further information regarding their overall infant feeding experience and behaviors, including breastfeeding and complementary feeding, related to responsive feeding methods and weaning.

**Results:**

Three themes emerged from the interviews—Human Milk Contents, Nurturing Qualities and Hunger/Satiety Cues, while the focus groups generated seven themes—Human Milk Attitudes, Weaning Age, Weaning Foods, Introduction of New Foods, Mealtimes, Crying/Fussiness Response, and Sweet Food/Candy. While, to the best of our knowledge, responsive feeding practices are not taught in Samoa, some of the themes highlighted responsive feeding practices already being utilized in Samoan families.

**Discussion and Conclusion:**

The information gained from the interviews and focus groups can be used to develop evidence-based messaging that strives to educate parents and caregivers to successfully identify hunger and satiety signals in infants and encourage the introduction of healthy and age-appropriate foods that complement current Samoan breastfeeding practices and childhood dietary patterns.

## 1 | Introduction

Promoting exclusive and then extended breastfeeding remains a priority in most global settings with broad benefits for both mothers and infants. Samoa—a low-middle income nation in the Pacific—has a strong history of breastfeeding promotion and protection (Ministry of Health, Samoa 2024; Soti-Ulberg et al. 2020)—with rates of initiation and exclusive breastfeeding to 6 months of age often markedly higher than its peer nations (Neves et al. 2021). While breastfeeding initiation is high in Samoa at almost 90%, only 56% of mothers continue to the recommended 6-months of exclusive breastfeeding (Samoa Bureau of Statistics 2021), and there remains an opportunity to explore factors associated with early weaning from breast milk and the introduction of complementary feeding to further strengthen local promotion efforts.

Previous research in Samoa has largely focused on structural barriers to breastfeeding—such as the need to return to work or the length of maternity leave (Archer et al. 2017; Arslanian et al. 2021; Daiy et al. 2023). In the context of questions about potential barriers to breastfeeding, mothers also raised beliefs about their milk “transferring good and bad factors of their diet to their offspring.” In some cases, the belief that the content of their human milk could be damaging to the baby led the mothers to supplement with formula or stop breastfeeding all together (Arslanian et al. 2021). This phenomenon was, however, only briefly explored in the prior research and there is still little known about how Samoan mothers perceive human milk composition, the role of milk quality, complementary feeding, and how breastfeeding promotes infant satiety—all of which may be contributing to the decrease in breastfeeding prevalence in the first 6-months of infan’s life in Samoa.

With the focus of most research on promoting breastfeeding, there has been little attention paid to the weaning process and when/how Samoans introduce solid foods—defined as any food other than human milk—into the diets of their infants (WHO 2023b, 2025). This is important, since early childhood feeding strategies have been shown to play an important role in establishing and regulating healthy eating habits and satiety responses, influencing healthier weight gain trajectories and reducing later obesity and noncommunicable disease risk (Brown et al. 2017; Hurley et al. 2011; Vazir et al. 2013; Worobey et al. 2009); challenges that are common among Samoan adults and children (Lameko 2022; Lin et al. 2017). In particular, research has shown the importance of encouraging responsive feeding when introducing solid foods into the infant diet (Fernandez Rao et al. 2020; Pérez-Escamilla et al. 2021; Worobey et al. 2009).

The theoretical background for responsive feeding originated in anthropology, psychology, and nutrition—grounded in behavioral reciprocity between parent and child alongside a responsive parenting style that is culturally mediated (Black and Aboud 2011). Anthropological research has found that culture influences all aspects of the infant weaning process and subsequent responsive feeding practices, including the when, how, and what of introducing solid foods (Cassidy and El Tom 2014; Dettwyler 2004; Quinn et al. 2023; Tomori et al. 2017). Anthropologists often explore cultural variation in infant feeding practices, breastfeeding and weaning ecology, biocultural lactation, mother–infant interaction patterns, and the social aspects of feeding, which provide key context for responsive feeding concepts (Gray 1996; Little et al. 2018; Martin 2017; Miller et al. 2025; Quinn et al. 2023; Tomori et al. 2017; Vitzthum 1994; Vitzthum and Aguayo 1998). These cultural contributions highlight the need to study under-researched populations such as Samoa in order to fully understand the cultural contributions to responsive feeding variations seen globally.

The fundamental principles of responsive feeding include parental understanding of infant hunger and satiety signals, recognizing readiness to introduce solid foods that complement breastfeeding practices, and responsive approaches that expose infants to a variety of healthy and developmental stage-appropriate foods (Pérez-Escamilla et al. 2021; Savage et al. 2018). For the infant, responsive feeding practices promote an interest in eating diverse healthy and nutritious solid foods and transition him/her to independent eating, with attunement and understanding of their developing hunger and satiety cues, and the ability to successfully communicate these internal cues to their caregiver (Black and Aboud 2011; Hurley et al. 2011). Responsive feeding guidelines have been developed that are designed to introduce infants to solid foods that complement extended breastfeeding practices and positively impact child feeding behaviors and self-regulation and later weight outcomes (Black and Aboud 2011; Engle and Pelto 2011; Pérez-Escamilla et al. 2021).

Caregiver and family practices play an important role in successfully implementing responsive feeding behaviors. Mealtimes together with family and caregivers—as opposed to feeding the infant alone, separate from other children or adults—have been shown to promote the development of responsive feeding and optimal eating behaviors (Hammons and Fiese 2011; Pérez-Escamilla et al. 2021). This is believed to be due to the ability for others to model or encourage healthy eating patterns through modeling optimal behavior (Casazza et al. 2015; Engle and Pelto 2011). Additionally, guidelines for responsive feeding recommend that the feeding environment include nurturing behaviors free of distractions such as screens—such as positive verbalization, encouragement, patience, and the avoidance of forcing or coercing infants to eat (Engle and Pelto 2011; Pérez-Escamilla et al. 2021; Savage et al. 2018; Vaughn et al. 2016). How parents interpret and respond to signs of fussiness and/or crying can impact the successful implementation of responsive feeding recommendations. Information on responsive feeding in Samoa, such as maternal knowledge of responsive feeding practices and implementation during the infant weaning period, defined here as the period in which complementary foods are introduced, has yet to be explored in current literature.

Building on the previous research in Samoa and attempting to address the knowledge gaps cited above, we conducted a two-part, longitudinal mixed-methods study in Samoa. First, we conducted structured interviews with mothers of infants aged 2–4 months to capture mothers at the point that they may have begun thinking about introducing solid foods and explore knowledge of breastfeeding practices, human milk composition, and infant hunger and satiety cues. Then, a subset of the sample was asked to participate in focus groups approximately a year later to retrospectively explore their experiences of weaning and the use of responsive feeding practices at the individual and family levels. The overall aim of the study was to understand maternal experiences of human milk, breastfeeding, and weaning and to determine gaps in knowledge or education around responsive feeding, breastfeeding, and the introduction of solid foods that may be addressed with future interventions.

## 2 | Methods

This research was conducted as part of a larger study focusing on hormonal and microbiome transfer through human milk in maternal and infant dyads alongside maternal and infant body composition. The primary study collected maternal and infant anthropometric data, human milk samples, and questionnaire data in addition to the interviews and focus groups. A brief overview of the methodology specific to the research question addressed in this article is provided below.

### 2.1 | Ethical Review

Participants gave their informed, written consent for all study procedures. This research received human subjects approval from both the Yale University Institutional Review Board (#2000028425) and the Samoan Ministry of Health (MOH) Health Research Committee.

### 2.2 | Population and Setting

This study was conducted at the Obesity, Lifestyle, and Genetic Adaptations (OLaGA) research center, situated in the Samoa MOH building in the capital city of Samoa, Apia. For the initial study (2020–2021), we recruited a sample of *n* = 100 mother-infant dyads who were exclusively breastfeeding at the time of data collection. To be eligible for participation, the participants had to be aged 18–45 years, of Samoan ethnicity, and exclusively breastfeeding a singleton-born infant age 2–4 months (±1 week) at the time of data collection. Exclusion criteria included a history of diabetes (Type I, Type II, or gestational) and smoking during pregnancy. Full inclusion/exclusion criteria can be found in Bertacchi et al. (2025). Recruitment was limited to those residing in Upolu, the smaller but more populated of the two islands that make up Samoa. Convenience sampling was used to recruit participants via advertisements on social media and posters placed in antenatal and well-child clinics across Samoa. Participants who expressed interest in being considered for the focus groups during the initial recruitment wave were recontacted 1 year later. For the focus group subset, we were able to recruit *n* = 25 of the initial study group. The focus group participants were split into five groups based on their availability.

### 2.3 | Study Design

**FIGURE 1 |:**
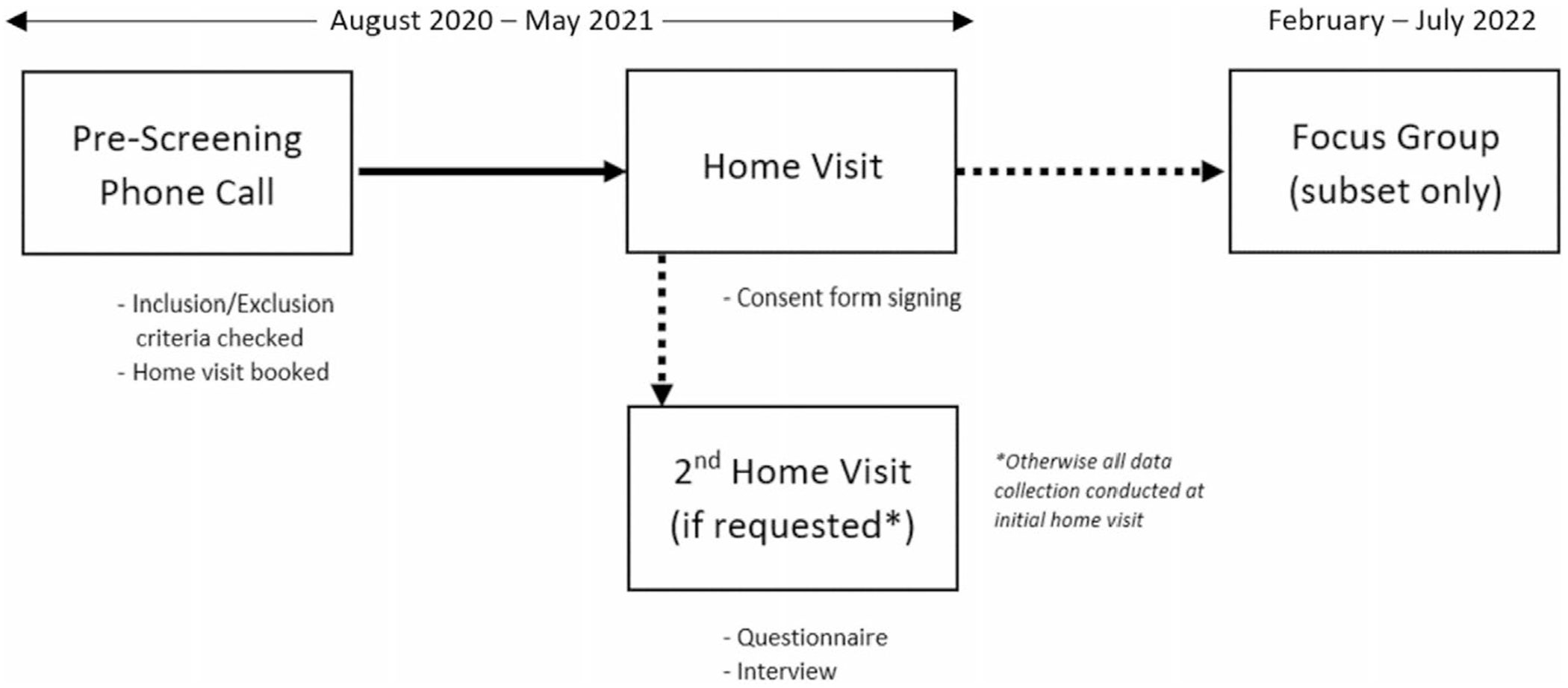
Study timeline.

The larger study was conducted through a combination of home visits and a visit to the research center made by the participant. The research for this paper includes data from questionnaires and structured interviews conducted at the home visits as well as focus groups with a subset of the participants a year later. Participation in the study pertinent to this paper involved the following steps (in order; Figure 1): (1) a pre-screening phone call, (2) a home visit for consent form signing, (3) a second home visit, if requested by the participant, and (4) a focus group a year later (subset only).

#### 2.3.1 | Questionnaire

All participants completed a questionnaire at 2–4 months post-partum that included: (1) a demographic questionnaire that collected information such as age, marital status, educational status, employment, average income, and parity, and (2) an adaptation of the Infant Feeding Practices Study II survey (Fein et al. 2008) that collected information on infant feeding practices, such as average frequency and duration of breastfeeding bouts per day. Questionnaire data was collected and managed using REDCap electronic data capture tools hosted at Yale University (Harris et al. 2009, 2019).

#### 2.3.2 | Interviews

One-on-one structured interviews were conducted by Samoan research assistants using open-ended questions to encourage participants to express their opinion on the topics presented. Interviews were conducted in the participants’ homes during the Home Visit. The interview had two objectives: (a) to gain an understanding of human milk knowledge in Samoa, particularly opinions on the content of human milk, worries about human milk quality, and what is being passed from mother to infant via human milk, and (b) to explore maternal knowledge on infant hunger and feeding cues (infants aged 2–4 months). Specifically, we were interested in opinions on breastfeeding quality, how mothers gauge infant cues of hunger or fullness, and where this knowledge has been gained. The interviews were short in length—lasting approximately 5–10 min on average—designed with the intent to gather themes to be further explored in the later focus groups. Each participant was asked the same questions to allow for comparison across the sample. If necessary, the interviewer would re-word or clarify a question if the interviewee expressed confusion. The interview questions can be found in the Supporting Information (Supplemental 1).

#### 2.3.3 | Focus Group Interviews

The five focus groups, completed between February and July 2022, also utilized a semi-structured approach, encouraging discussion among the group. While the moderator would commence the conversation by introducing the topic of interest, focus group participants were encouraged to engage in open conversation and interact with each other throughout. The focus groups were conducted in local meeting areas that were convenient for the participants. Questions from the individual interviews that had been asked of participants at 2–4 months postpartum about hunger and satiety cues were repeated in the focus groups to explore changes in maternal perception and opinion over the 1-year timeframe. In addition, further questions focused on topics of responsive feeding methods, timing of supplementary food introduction, structure of family meals, and inquiry into where they acquired knowledge to guide their feeding practices. The focus group agenda—with sample questions—can be found in the Supporting Information (Supplemental 2).

All interviews and focus groups were conducted by two female native-speaking Samoan Research Assistants in Samoa. The Research Assistants were both trained and had experience conducting qualitative interviews and moderating focus groups. Interviews were voice-recorded to allow for later transcription, translation to English, and qualitative analysis.

### 2.4 | Data Analysis

Transcriptions from the participant semi-structured interviews and the focus groups were coded separately in NVivo software (NVivo 12 Pro) utilizing an inductive approach to identify key themes. An initial coding scheme was devised based on the interview questions, with subsequent codes added as they arose throughout analysis. Once all interviews were completed in the initial pass, they were re-coded until no new codes emerged, indicating saturation was achieved. To reduce researcher bias, VB conducted the initial round of coding and KD checked the codes—disputes were resolved by a third researcher (KL). Themes were subsequently explored using thematic data analysis tools in the NVivo software, in which individual codes were read in aggregate and finally a written summary of each code was produced. The breakdown of thematic coding can be found in the Supporting Information (Supplemental 3). Saturation was reached for each theme, based on the emergence of no new codes on the final iteration of coding and similar responses across interviews.

## 3 | Results

### 3.1 | Demographic Characteristics of Study Participants

Mean maternal age was 27.7 ± 6. 2 years and mean infant age was 2.7 ± 1.0 months (Table 1). Most infants were female (63%). Most characteristics of focus group participants were similar compared to the larger study group with no statistical difference between the two samples, suggesting a representative subset at enrollment. However, although not statistically significant (*p* = 0.3 and *p* = 0.5, respectively), there were differences in the distribution of infant sex and region of residence.

While the residence of Apia Urban Area is similar between the two samples (~20%), the distribution of the other census regions differs with lower residence of peri-urban Northwest Upolu region (subsample; 40% vs. initial sample; 53%) and higher residence in the rural rest of Upolu region (subsample; 40% vs. initial sample; 27%).

### 3.2 | Infant Feeding Questionnaires

The questionnaires were given to all participants (*n* = 100) during the initial study phase. A significant proportion of the participants stated they practiced breastfeeding on-demand (45%) with breastfeeding bouts lasting 30–39 min (41%) (see Table 2). The great majority of participants (88%) felt confident in recognizing if their breastfeeding infant was receiving enough milk. Only 39% of the participants stated that someone had explained to them how to recognize if their infant was receiving enough milk or not. Of those 39%, when asked in an open-ended question from whom they received information regarding infant satiety, 71% stated their own mother as their primary resource—of which almost half stated a combination of own mother and their doctor.

### 3.3 | Interview Themes

The semi-structured interview addressed human milk composition, what is passed from mother to infant while breastfeeding, and maternal perception of infant hunger and satiety cues. From the interviews, 23 codes were identified, and these were combined into 3 themes—Human Milk Contents, Nurturing Qualities, and Hunger/Satiety Cues.

#### 3.3.1 | Human Milk Contents

The majority of respondents (68%) directly associated what the mother eats and what is in her milk. Many participants stated that all food consumed changes into human milk to be given to the infant and therefore stressed the importance of maintaining a healthy diet themselves.

I think it’s the food we consume that produces the breast milk like green veggies and heaps of starchy food like taro and the more you eat those foods the better the breast milk you have.

PPT 93, 31 years old, 3-month-old female infant, 3^rd^ baby

Everything I eat, for example, I eat laupele so I’m passing that vitamin to my baby even the fat that I eat I’m passing that to my baby as well.

PPT 100, 26 years old. 3-month-old female infant, 3rd baby

The most common foods quoted as being good at producing human milk were vegetables—in particular leafy greens such as laupele (variety of spinach) and cabbage (74%), liquids such as soup or coconut water/milk (56%), starches such as taro (29%), fruits—in particular banana and pawpaw (27%), and lastly fish and chicken (19%).

The second most common response (31%) for the contents of human milk described biological components—for example vitamins, minerals, protein, and water, though it should be noted that these were often also linked back to the diet (10/31). For example:

[…] my understanding about what’s contained in breast milk is that the food that I’m eating produces breast milk, it has vitamins and proteins and minerals for the baby to be strong and healthy.

PPT 23, 35 years old, 4-month-old male infant, 3rd baby

[…] I think nothing beats breast milk, it has vitamins, proteins from the food I eat so that my baby can be healthy and strong, so breast milk is the best for my baby.

PPT 43, 25 years old, 3-month-old male infant, 4th baby

My understanding is the vitamins from the food I eat, and I need to eat lots of vegetables for my baby to be healthy.

PPT 99, 26 years old, 3-month-old male infant, 3rd baby

#### 3.3.2 | Nurturing Qualities

When asked what is passed from mother to infant through human milk, the main responses were bond/love (58%), health (48%), protection from diseases and illnesses (28%), and strength (24%). The vast majority of the participants conveyed no concerns or worry in terms of milk quality (98%). The two women who expressed concern over the quality of their milk stated reasons relating to their own diet and intake of healthy food.

Yes, because sometimes I dont’ eat healthy food but if I eat healthy food then I’m not worried at all.

PPT 76, 30 years old, 1-month-old female infant, 4th baby

Yes, I feel worried when I don’ eat healthy food and then I breastfeed my baby, I’m worried because she might get a sore stomach.

PPT 79, 26 years old, 3-month-old female infant, 1st baby

#### 3.3.3 | Hunger/Satiety Cues

Hunger cues were commonly cited as infant crying and fussiness (100%), placing fingers in mouth (51%), feelings of fullness in breast (20%), and rooting for the breast (9%). The most common satiety cues were turning head away from the breast/no longer wanting to feed (85%), falling asleep (47%), calmness (25%), and no longer crying (20%). When asked at what age they were able to identify the hunger and satiety cues in their infants, on average participants noted learning their infant’s signs for hunger earlier than those associated with satiety (1-month vs. 2-months postpartum, respectively).

### 3.4 | Focus Group Themes

**FIGURE 2 |:**
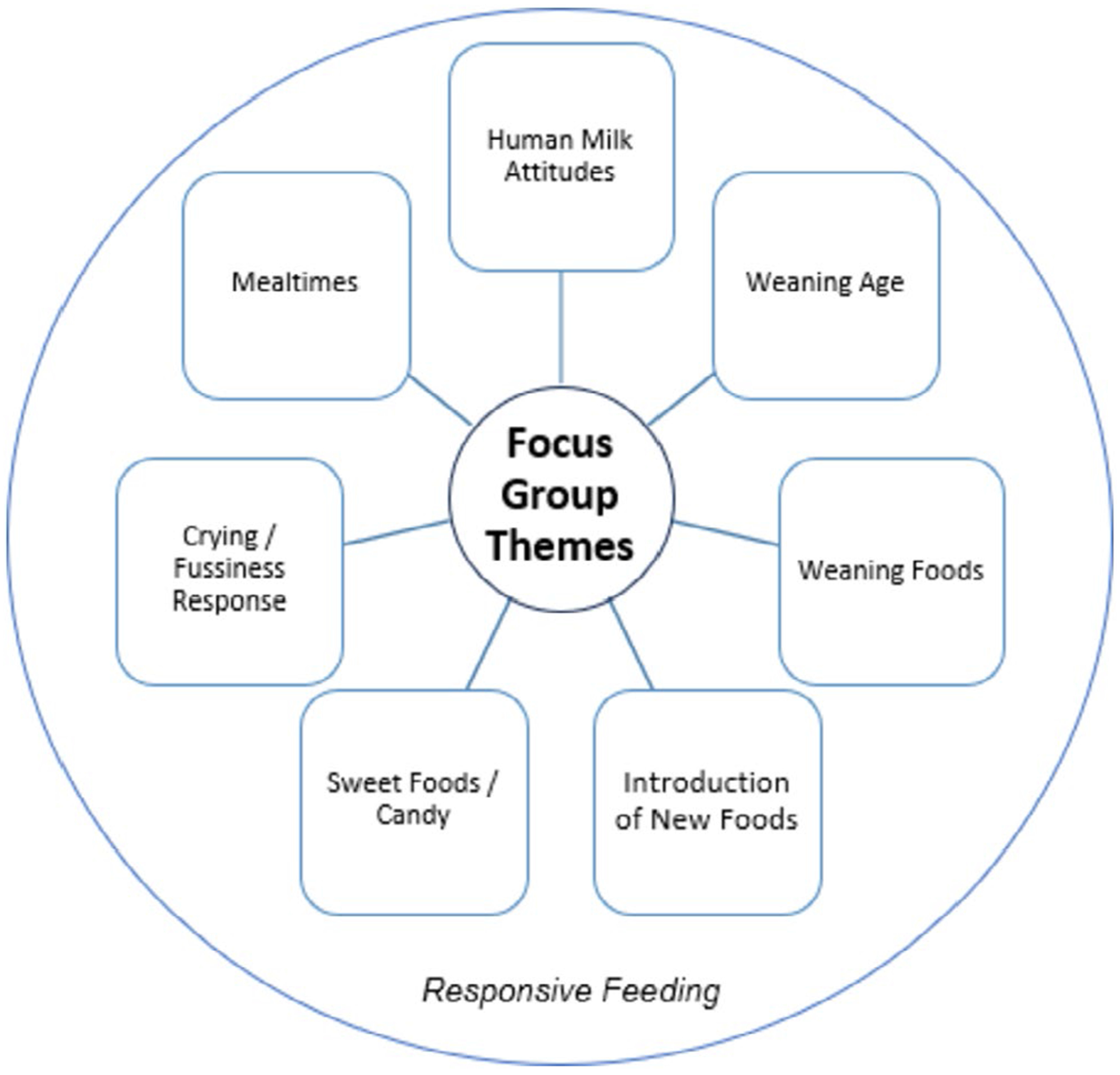
Focus group themes.

The focus group interviews addressed what foods are given to different ages from birth to age 2, the weaning period, responsive feeding, mealtime structure, and how mothers respond to infant crying and/or fussiness. 108 codes were identified, and these were combined into 7 themes—Human Milk Attitudes, Weaning Age, Weaning Foods, Introduction of New Foods, Mealtimes, Crying/Fussiness Response, and Sweet Food/Candy. The last theme (Sweet Food/Candy) interweaves with the other 6 themes. Additionally, responsive feeding behaviors emerge throughout all identified themes. A visual breakdown of the themes is presented in Figure 2.

#### 3.4.1 | Human Milk Attitudes

All focus groups mentioned that human milk is the best food for infants under the age of 6 months. When asked to explain why and what the benefits of human milk are, the main responses discussed were protection against disease and sickness, the biological components, how it makes the infant healthy, how it makes their infants strong, and the ease—mainly cost and convenience.

Well I would like to share that the only person who first taught me how to care for a baby was my grandmother. She helped me a lot but when she died in 2014, that’s when I was able to care for my own, by preparing healthy food in order to have good breast milk for the baby. Because she said that if I eat healthy food, I will be happy, which means that when my baby eats from the breast milk that I am producing, she will also be happy. So until now, my kids are happy which tells me that breast milk is very important and that I should feed and breastfeed my kids every day.

Focus Group 2 Participant: 30 years old, 4 children, Apia urban area

The two most common challenges to breastfeeding that were mentioned were what the mother eats and breast pain and/or leaking. Less commonly mentioned were biting, insufficient milk quantity, and needing to return to work. All groups referenced that what the mother eats is directly associated with the quality of their human milk. This had both positive and negative connotations depending on how the participant viewed the quality of her own diet. For example:

Well, my only saying is that, I am well familiar with breast milk as I have lots of children, breast milk is very important if the mother is well prepared and clean with cooking food to eat, it will also give healthy breast milk for the wellbeing of the baby. So every time when the baby is breastfed, it’s like all the strength of a mother goes with it as well so that the baby becomes stronger and healthier. She should keep on eating soups, vegetables, porridges and all those kinds of food that provides a healthy body and mind for the baby because of this breast milk from the mother. That’s my own opinion about the importance of breast milk.

Focus Group 2 Participant: 30 years old, 4 children, Apia urban area

To me, I always had a hard time breastfeeding my baby because of the kinds of food that I eat. Instead of eating food that makes you stronger but eating fish and chips and things that I love eating and forgetting the fact that I am also breastfeeding my baby. So no matter how hard the baby tries to suck there’s no breast milk because of me and the things that I am eating. I am not sure about others but those are some of the facts that I am now having regarding my breast milk.

Focus Group 2 Participant: 39 years old, 6 children, Apia urban area

Discussion of hunger cues replicated the earlier interviews and were commonly cited as infant crying and fussiness, feelings of fullness in breast, and placing fingers in mouth. The most common satiety cues were falling asleep, turning head away from the breast/no longer wanting to feed, and biting/playing with nipple. When asked what changes with age in regards to understanding hunger and satiety cues for infants, the two main discussions were communication either by speaking, pointing, or crawling toward the breast.

#### 3.4.2 | Weaning Age

**FIGURE 3 |:**
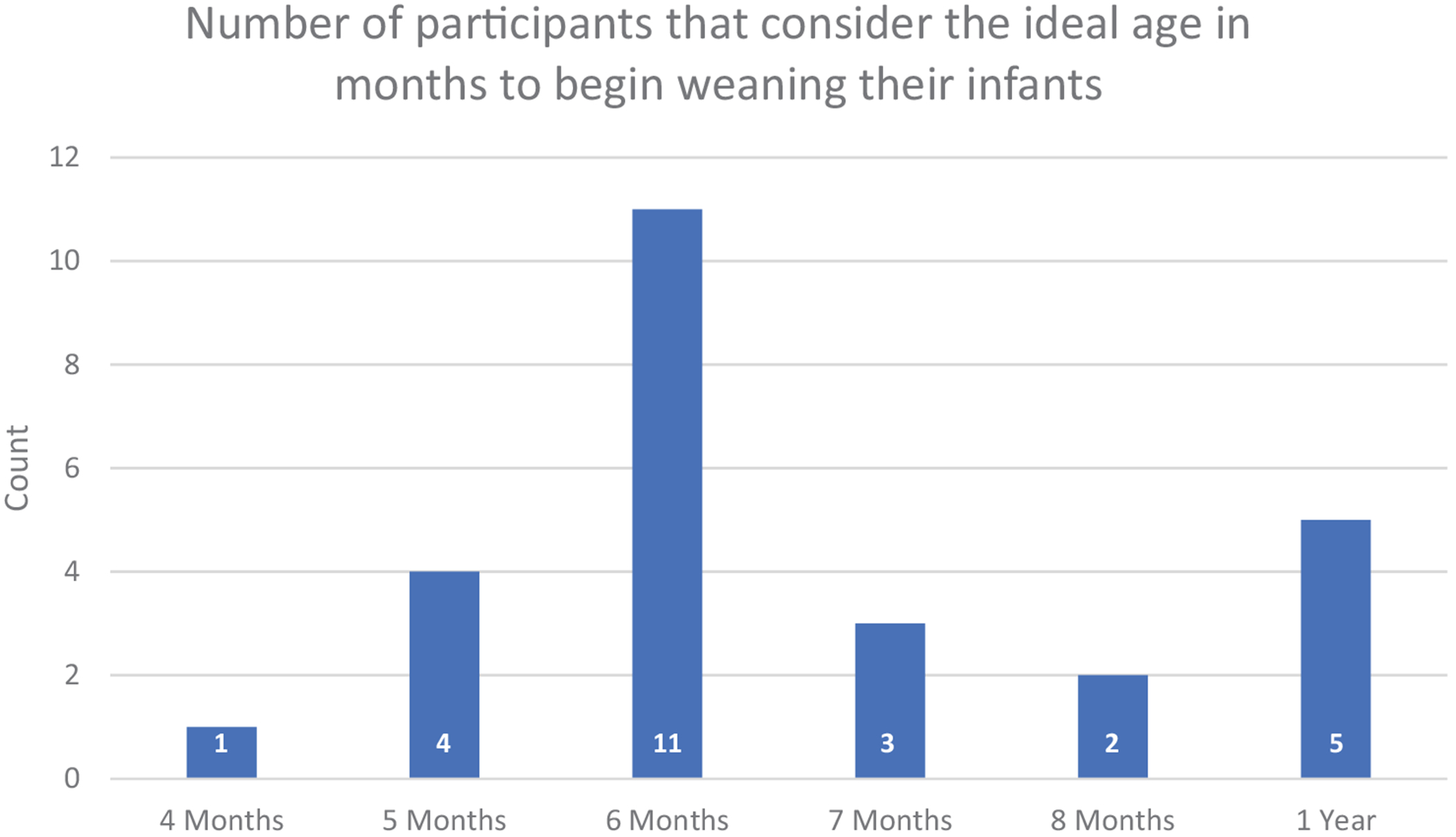
Participant opinion on ideal age of introducing solid foods to infant diet.

The term “weaning age” in this context is used to describe the age at which solid foods are first introduced into the diet, detailing the marker for when milk is no longer the sole source of dietary intake. Across the focus groups, the age at which to introduce solid foods was debated among the participants. Consensus was split across the first year of life with the divide as follows (see Figure 3):

All groups mentioned their own parents—particularly their mother—as the main source of knowledge for when and how to start weaning their infants. The other sources of knowledge were the hospital staff, the Ministry of Health (MOH), TV, and the vaccination card (the Samoan MOH includes breastfeeding education on the vaccination card). When queried on how to tell if an infant is ready for starting solid foods, all groups mentioned the infant having teeth as an indication of readiness, alongside demonstrating the ability to chew and showing interest in foods other than human milk.

#### 3.4.3 | Weaning Foods

A minority of the participants stated that they introduced solid foods to their infants under the age of 6 months, though all emphasized that human milk was still the main source of nutrition. When asked what foods they introduced, the lists highlighted soft or liquid foods such as porridge, soups, juices, and papaya.

My understanding about babies 6 months old and under, breast milk was the only suitable food for them. But for me, I usually feed my kids when they are 5 months old, even if they are breastfeeding, I used to feed my baby with potatoes which goes together with the breast milk.

Focus Group 2 Participant: 30 years old, 4 children, Apia urban area

At the time we give birth, they say it should be the best to breastfeed the baby, even at 4 months when you can feed the baby with solid food. We used to feed them with food that contains sago [tapioca pearls] and porridges but also breastfeeding so that the baby can have a strong body without getting sick.

Focus Group 1 Participant: 21 years old, 2 children, Northwest Upolu

Well my own understanding about it, the mother’s breast milk should be the only thing that we should feed the baby with. Like at 5 months, the baby should also be feeding with liquid foods like local spinach. Boil the spinach until it’s boiled, then use it to feed the baby. It is also useful for the mother to eat it as it is good for producing more breast milk for feeding the baby so that the baby can have a healthy life.

Focus Group 2 Participant: 39 years old, 6 children, Apia urban area

More commonly discussed were the foods that were given to wean infants between 6–12 months of age. The foods discussed fell primarily into three categories—soft/liquid, sweet/sugary, and healthy. Examples of soft or liquid foods included: soup—of particular mention was pumpkin soup (21%), spinach (18%), taro (14%), porridge (13%), meat/fish (11%), fruit—mostly banana (10%), and potatoes (10%). Other items mentioned included rice, softened vegetables, and coconut milk/cream.

I would like to add with her opinion, yes not only those kinds of foods. There is also pumpkin, you can boil it until softened, smash it well and then feed the kid or another thing you can use is sago [tapioca pearls]. Those are kinds of soft things that you can use to feed the baby. For me, I feed my baby from 5 months going to 6 months, because it is my first baby so it’s like a new thing to me, but my mother was there for me. I firstly fed my baby with sago and then during her 7 months I started to feed her more with pumpkins, liquid foods that are good for the kid’s health.

Focus Group 2 Participant: 26 years old, 1 child, Northwest Upolu

Many of the participants mentioned sweet or sugary foods as first weaning foods, with some commenting that they were either easier for the infant to accept or that they were necessary at this age. For example:

So the kind of food that I know we should give to a baby of 6 months old to 1 year old, like soups that has vegetables in it is the most suitable food for babies, water and also fruits like banana, you can make a smoothie or an apple, so that the baby can eat it in order to gain strength […] Those are the things that we need to look at, but eating soups and drinking water is healthy but there should also be a sweet food for the baby’s body or a fruit because it is important for the baby’s body to have a bit of sugar in the body.

Focus Group 1 Participant: 21 years old, 2 children, Northwest Upolu

Of interest, when giving examples of sweet or sugary foods almost half of the mentions included biscuits as the foods that should be given to infants 6–12 months as their introduction to solid foods. Often it was emphasized how biscuits are easier for the infant to eat compared to harder foods. The term biscuits here refers to arrowroot and/or milk biscuits/cookies that are marketed for infants. Arrowroot is a grain-free starch that is often used as a base for infant biscuits and/or cookies.

From 6 months to 1 year old baby, to me and my understanding about the kinds of foods that the baby should eat like biscuits, if I gave a biscuit to her, it’s not like I get to give her any snacks but instead of biscuits and those kinds of things that we can put in the hand then she can chew because those kinds food can easily be eaten by the baby.

Focus Group 2 Participant: 39 years old, 6 children, Apia urban area

In contrast, those that mentioned an emphasis on healthy food often contrasted this idea against sugary or sweet foods and drinks. For example:

I know that everyone knows, those advertisements from the tv about advice from the ministry of health. They encourage us about healthy foods. There were also stamps that I’ve seen about not allowing kids to drink sweet drinks but instead water. Those are all the things they told so that there will be nothing that could harm the kid.

Focus Group 2 Participant: 18 years old, 1 child, Apia urban area

As with breastfeeding information, all groups mentioned their own parents as the main source of knowledge for what foods were best for introducing to their baby when weaning. Additional information came largely from the vaccination card, followed by the TV and then the MOH.

#### 3.4.4 | Introduction of New Foods

The main foods cited as being easy for infants aged 6–12 months to accept when first introduced were those that are sweet or treats—for example biscuits or candies. Other foods that were mentioned were soup, fruit, porridge, or taro. When asked what foods were difficult for infants to accept, every focus group discussed foods that are too hard in texture.

When discussing how to tell if an infant does not like a new food the three main signs were wasting the food (e.g., refusing to eat it), spitting out the food, and eating all other food offered except that new food.

I know when my baby is not happy by his facial expression, like the time when I feed him. I know very well when my baby does like the food I’m making, when he eats all of it. But if there’s food that doesn’t taste good, he won’t eat it no matter how hard I try to feed him.

Focus Group 1 Participant: 21 years old, 2 children, Northwest Upolu

So I know that those are the foods that the baby needs to eat but if you feed the baby some different food or the foods that the baby doesn’t want to eat the baby split them out or vomits, then I realize that my baby does not want the food that I served.

Focus Group 4 Participant: 22 years old, 3 children, Northwest Upolu

The discussion for how participants reacted to their infants’ response to new foods during this timeframe presented many subthemes that are prominent in responsive feeding behaviors and the promotion of optimal feeding practices. When an infant did not like a new food, the participants said they would react by either giving a different “safe” food instead, keep trying to familiarize the infant with the new food through repeated exposure, or offering the unwanted food in a different way.

[…] That’s the best time for me to sit down and see my children and their foods, what foods make them happy. If they don’t like the food, we’ll try it next time. For example, if this is the supoesi [papaya soup] that has been done, but the porridge was done yesterday […] but my children don’t like it, I’m tired of giving it to them because they don’t like it. It means that they don’t want it and I think that I should repeat the dish that they like. My baby who is 2 years old now, she only likes the one dish because it was the first food she was fed.

Focus Group 3 Participant: 35 years old, 3 children, Northwest Upolu

Some kids when given new foods, they just spit it out when they don’t like it. You also know those kinds of foods that the baby doesn’t like, then you can think of another way to do it.

Focus Group 1 Participant: 20 years old, 1 child, Rest of Upolu

When an infant did like a new food, the participants stated that they would then make it available to the infant more often and encourage him or her to try it in different styles or consistencies.

If my baby likes the food that I give him, it is my responsibility to do it in different ways. For example, like taro. My baby likes eating taro. I can also change the way of cooking it by putting it in a soup because it can be eaten together with other foods inside the soup.

Focus Group 1 Participant: 21 years old, 2 children, Northwest Upolu

The discussion around how to help encourage the acceptance of new foods fell into two categories: approaches that support responsive feeding and those that do not. For approaches that support responsive feeding, these included familiarizing, encouraging, repeated exposure, not forcing, and persistence.

My own understanding of the kids only depends on the mother. I have to try from Monday to another Monday, or from Sunday till the next weekend, at least that I am trying to maintain his meal. So from that you will notice that the kid is now familiar with those kinds of food throughout the whole week. That is one way you can teach the kid how to eat healthy foods because the reason why the kids eat sweets is because the mother gave it to them. The kid won’t be able to get those kinds of food from the stores, it all depends on the mother. That is my own understanding about having a new food.

Focus Group 1 Participant: 20 years old, 1 child, Rest of Upolu

Approaches that were not aligned with responsive feeding included force, punishment, and bribery.

The only thing you can do is by giving it to them instead of forcing them to eat. But in some ways it is also a good thing to force them to eat those healthy foods, so that when they grow up, they will be able to eat those kinds of food on their own. For example, my sister’s kids. I saw the way she treats her kids, meaning her kids love eating sweets. So she gave them a plate of fruits to eat. Unless those fruits are finished, they can never eat any other kind of food that they need to eat. So that is another way to teach a kid before growing old. It is better to teach the kid while at a young age, by the time they grow old, they are happy to eat healthy food like fruits then can eat a sweet later.

Focus Group 1 Participant: 22 years old, 1 child, Rest of Upolu

When discussing introducing new foods, the use of punishment was mentioned as a method either to discourage fussiness or to deter from eating sweets and candy. For example:

Just try to make the baby familiarized but the best way is to give them a little smack. Because some kids like to eat sweets, just to scare them. Because other kids like eating sweets when they reach 6 months old. So it is up to us because some kids, they don’t like eating vegetables, so we have to try to find a way to make them eat the vegetables, if not, a little smack will help.

Focus Group 1 Participant: 21 years old, 1 child, Northwest Upolu

For my response to that question, for most children those are the foods that they really like, sweet foods. They really like to eat any kind of sweet foods. My kids their father always give those sweet foods. Or give them the money to go buy foods from the shop like candies. But me I will not do that. If they crying for that I will tap them with the broom. But I will not give them sweet foods […].

Focus Group 5 Participant: 27 years old, 4 children, Rest of Upolu

#### 3.4.5. | Mealtimes

**FIGURE 4 |:**
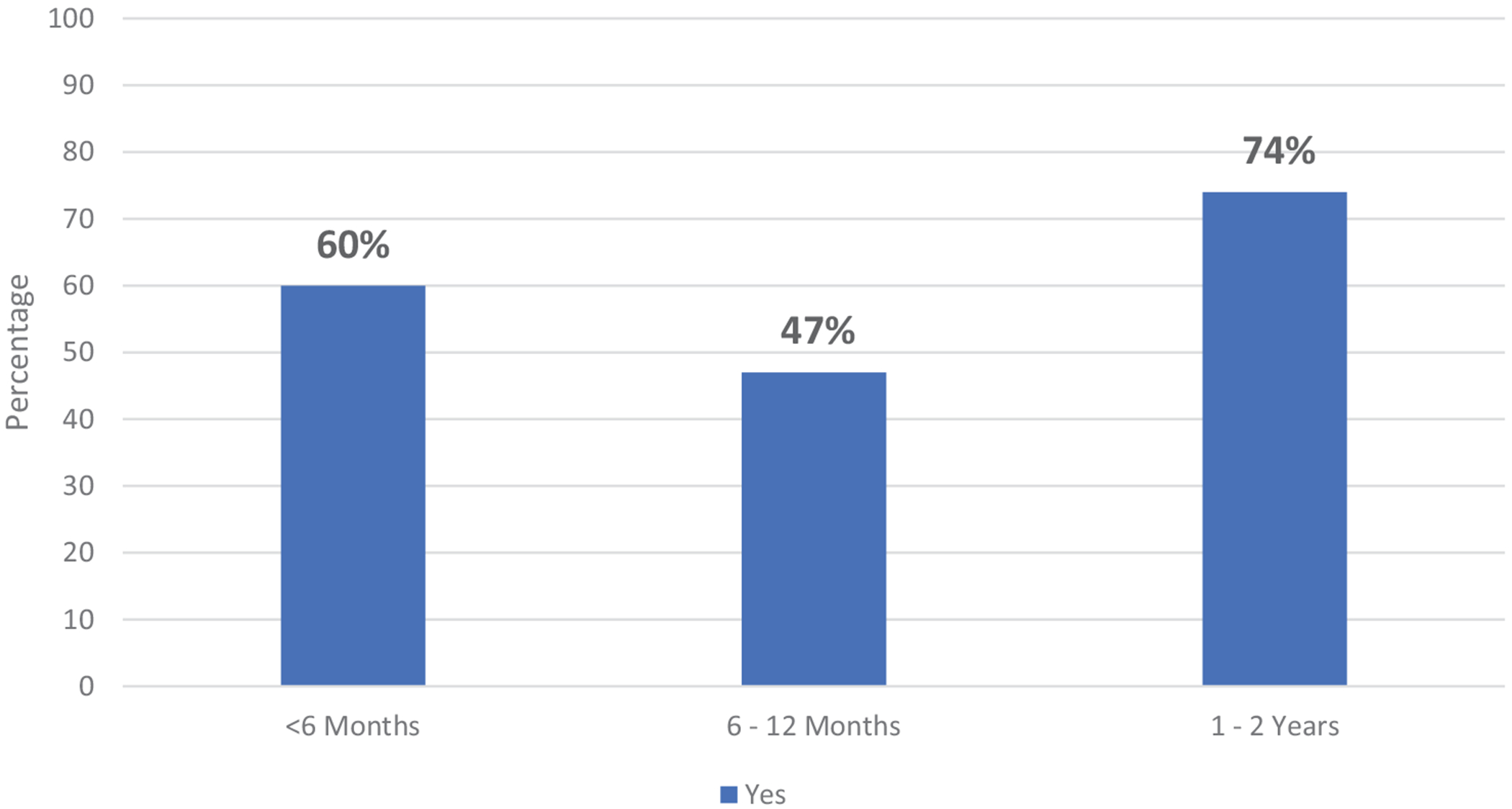
Percentage of responses that state inclusion of infants of different ages at family mealtime.

Participants were asked whether infants of certain ages (< 6 months, 6–12 months, and 1–2 years) were included in mealtimes at the table with the rest of the family at that particular age. See Figure 4 for distribution of responses.

The participants that included children at the table often cited that mealtimes were for the entire family to participate together, and many emphasized that no technology—such as television or electronic screens—was allowed while eating. Both of which are methods used to promote responsive feeding behaviors. For example:

The family of my child is very strict, yeah, they have a television but they are still straight up to the old system, they don’t turn on the television at the mealtime. In my family they have a breakfast, lunch, and dinner in my family on the time of mealtime, even the time that I have a baby, when we have a dinner, we all eat at that time. We eat together because we want to teach them and let them understand but the other family they don’t have a time together with children. Well that was our routine in my family, they give their priority to children and also there’s no one watching television on the time of meal, even telephones. And I know that we all eat together because we have an oldest Mama. She’s 90 years old and she really wants to be with her children every time on the table.

Focus Group 3 Participant: 35 years old, 3 children, Northwest Upolu

When it’s time to eat, everyone will eat together but as for my baby, she won’t like sitting next to me but instead beside her dad. So my husband will eat and feed her at the same time. As of now she likes to eat on her own and regarding the phone and the tv, I forbid my kids from those things because of what I saw nowadays. So if my kids came to me and said that they have seen other kids using phones or watching tv [while eating], I always tried my best to avoid them from doing these things.

Focus Group 3 Participant: 34 years old, 4 children, Rest of Upolu

The participants that specified that they did not include their infants at the table with the rest of the family during mealtimes stated reasons such as difficulty eating while holding a baby (< 6 months), infants are already asleep by the time older family members would like to eat dinner—this was especially cited for infants under the age of 1 year, and that they prefer for children to eat first before the rest of the family. For example:

My answer with that question, if the family have the meal at night, if the food is cooked, then the mother call to get the kid’s food first to be cold, so the kids will be feed first, so the whole family have their meal but the kids already ate they just watch tv.

Focus Group 4 Participant: 20 years old, 1 child, Rest of Upolu

In my family we always feed the kids first before the elderly came last. When my kids have their meal, sometimes I put their dinner table in front of the tv sometime I did not put it on the tv […] But for this time, in my family, this is what we always do. We always feed the children first and then the old people eat later. That’s how my family does for my kids.

Focus Group 5 Participant: 25 years old, 3 children, Rest of Upolu

One participant stated that infants under the age of 6 months cannot be included at the family table during mealtimes as it will make them want to eat solid foods before they are ready.

Just want to answer this question. Well I cannot bring my kids to the table because there is a disadvantage to it. Because if you bring your kids to the table during meal time, your baby will breathe the smell of food and that is the reason which makes your baby want to eat but you can’t feed your baby at the moment. That is my own understanding about it.

Focus Group 2 Participant: 26 years old, 1 child, Northwest Upolu

Participants that did not include children in family mealtimes often stated the use of technology to either calm their children in order for them to eat or as a distraction tool to be used while the rest of the family eats.

Like that is another way to calm the kid and me as well, that is an easy way. Because if I don’t give the phone for them to watch, the kid will not calm down as well or just run around and eat. He loves eating and watching, like that is another easy way to calm the kid and let him eat.

Focus Group 1 Participant: 20 years old, 1 child, Rest of Upolu

To my family, we eat around 4 o’clock. […] So I feed my two younger kids first then we have our evening prayer then the older ones eat later. After we eat, then they go watch the tv or they do their school work. Because to me and my children I feed them first then after eating then went to do their homework or watch tv. That is my plan for my family.

Focus Group 5 Participant: 35 years old, 7 children, Northwest Upolu

#### 3.4.6 | Responding to Crying/Fussiness

Participants were asked to discuss how they reacted to crying and/or fussiness from their infant and how this changed as the child transitioned from infancy (6–12 months) to toddlerhood (1–2 years). Responses were coded as either those that align or those that do not align with responsive eating practices.

When discussing their reactions to crying and/or fussiness in infants aged 6–12 months, all responses were conducive to responsive feeding practices. These included calming and/or comforting the infant, responding to their needs, and distracting them. For example:

It is the mother’s responsibility to take the baby in her arms when they are crying and breastfeed them. Try to calm the baby down because they are still not able to talk and tell us what he/she wants, but us, mothers, hold the baby in your arms, breastfeed, continue holding until the baby is calm and keep them warm. That is our job that needs 24 hours to be there beside your baby to monitor their every movement.

Focus Group 1 Participant: 21 years old, 2 children, Northwest Upolu

My kid is still very young, only 1 year old. He sometimes cries when needs a biscuit but if there is no biscuit, I would give him rice instead because it’s not that you will give them everything they would ask for. Because if they cried for something like that but it’s only you and your baby at home, then you don’t know what you are going to give to them. You wouldn’t know who will look after your baby while you are going to buy what they need, so I’ll just have to give them rice instead.

Focus Group 2 Participant: 18 years old, 1 child, Apia urban area

However, when discussing their reactions to cry and/or fussiness in infants aged 1–2 years, the responses were split between those that were conducive toward responsive feeding practices and those that were not. Responses that were conducive toward supporting responsive eating practices included discussion of techniques such as calming and/or comforting or identifying the problem. For example:

The ages of 1 to 2 years old, you can notice their attitude changing. You can easily notice the attitudes of your child since the time when they are still in your belly. Those are the kinds of behavior you can see in your kid, but it is the mom’s responsibility to control these behaviors so that the kid won’t easily get mad because there is a reason why they got mad hence why they throw something because there is something they need. So what I do was trying to calm them down and try to find out what the kid actually wants.

Focus Group 2 Participant: 39 years old, 6 children, Apia urban area

Whereas responses that were not conducive to supporting responsive feeding practices included discussions such as giving technology as a distraction or punishing/reprimanding. For example:

It is the same problem that is happening with my child. Sometimes she wants to change her meal or wants to play on the phone. I would firstly show her the stick to scare her off, but if I noticed that she’s still crying, then I would give her a bath, breastfeed her and put her to bed. If she continues crying then I will use a stick [to chase away] but if she wants to eat a biscuit, I would give her a slice of papaya instead because she likes eating papaya.

Focus Group 2 Participant: 30 years old, 4 children, Apia urban area

If my baby found a book then he is crying and wants to use it, then we have to look for a pencil and give it to him, but if my baby sees us holding a cell phone then the baby cries for ABC ABC like that […] I turn on the ABC song and then my baby watches. Sometimes I saw my baby moving his hands trying to copy the movements of the song.

Focus Group 5 Participant: 25 years old, 3 children, Rest of Upolu

#### 3.4.7 | Sweet Food/Candy

The theme of sweet foods and/or candies emerged often in conversation throughout the focus groups, though it was most evident when discussing which new foods were easiest to introduce to infants. For example:

All I know about the new foods that the kids started to eat, like cereals, or cookies that bought from the shops, just buy it and give to the kid’s hand, I know those are the new foods to give to the kids.

Focus Group 4 Participant: 22 years old, 3 children, Northwest Upolu

Some food that the kids really want to eat, if they don’t eat before like twisties, then they went to the shop, my baby crying for a twisty. The other mothers had good points, mostly my kids are always happy to eat sweet food. Sometimes I will give to them but sometimes I won’t, because I know it’s really bad for the kid’s health.

Focus Group 5 Participant: 25 years old, 3 children, Northwest Upolu

In addition, sweet food was also mentioned as a bribery tool to encourage eating and trying different foods—a practice that is contrary to the practice of responsive feeding. For example:

The only thing I know to keep the baby eat those new foods, I will sit down close to the baby and then I said come baby, look baby it’s yummy mmm yummy, those are the words that I have to say to my baby to persuade the baby to eat the food, or you can tell the baby it is delicious, come baby just try the food that mummy made for you, then I give the food to my baby or try to say something nice to the baby like after you eat your food then we go to the shop to buy you an ice-cream. All I want is for the baby to finish the food.

Focus Group 5 Participant: 35 years old, 6 children, Northwest Upolu

#### 3.4.8 | Responsive Feeding

Responsive feeding practices were interwoven throughout all the focus group themes above. For example, participants demonstrated responsive feeding behaviors when they identified nurturing responses to hunger and satiety cues, including techniques such as repeated exposure, familiarizing, encouraging, and not forcing when introducing new foods, and including infants in family meals. Nonresponsive behaviors included those where children were bribed with sweets/candy. While the topic of responsive feeding connects throughout all the themes presented in the focus groups, only one participant directly mentioned awareness of implementing responsive feeding.

So about this kid who was able to eat, my baby that I had right now, when she was able to eat, she started to eat less from the breast milk but more on the normal foods like soups. She knew when to stop which means that she’s full already when she pushed the plate away from her. She used to play with her food if I gave it to her for her to eat on her own.

Focus Group 2 Participant: 39 years old, 6 children, Apia urban area

## 4 | Discussion

This research used data from structured interviews (*n* = 100) and focus groups (*n* = 25) to explore maternal experiences of breastfeeding, weaning, and infant feeding. The interviews revealed three themes—Human Milk Contents, Nurturing Qualities, and Hunger/Satiety Cues and the focus groups revealed seven themes—Human Milk Attitudes, Weaning Age, Weaning Foods, Introduction of New Foods, Mealtimes, Crying/Fussiness Response, and Sweet Food/Candy. The topic of responsive feeding was found to weave throughout the focus group themes.

Participants were asked in both the individual interviews and the later focus groups to identify hunger and satiety cues that their infants exhibited. There was no difference in the hunger or satiety cues that were cited between the two time points, indicating that mothers are able to identify hunger and satiety cues in their infants early. However, in the individual interviews at 2–4 months postpartum there was a slight disparity in the self-reported timing of being able to identify hunger versus satiety cues in their infants. On average, participants noted that they learned their infant’s signs for hunger earlier than those associated with satiety (1-month vs. 2-months, respectively). As a key component of responsive feeding is recognition of infant cues and prompt response (Pérez-Escamilla et al. 2021), this may be a potential target for interventions to promote optimal responsive feeding practices.

A reoccurring theme across both the interviews and focus groups was that of human milk comprising of what the mother herself eats. It was often mentioned that healthy food makes for healthy human milk. Some participants saw this as a challenge to breastfeeding if they believed that their diet was not adequate enough to produce “healthy” milk. While the Center for Disease Control and Prevention (CDC) states that the maternal diet does not impact the quality of her human milk (CDC 2024a), research has found that many cultures believe that there is a link between maternal diet and human milk quality (for example: Poland (Karcz et al. 2021), Canada (Kidd et al. 2019), Korea (Jeong et al. 2017), South Africa (Chakona 2020), India (Chakrabarti and Chakrabarti 2019)). Interestingly, when discussing what types of foods they believed contributed toward “good” human milk, the foods cited by the participants were the same as those later cited as being the best foods to first introduce infants to. This could be of interest as a tool in advocating for maintaining a maternal healthy diet during the period of lactation, that hopefully would extend beyond the breastfeeding period.

In contrast to the healthy foods mentioned above, the food type cited as the easiest to introduce when weaning infants onto solids were sweets or candies. There were more mentions of this category of food than all other foods combined. In addition, sweets and candies were mentioned as a tool for bribing infants to eat and try different foods, which is a non-responsive feeding practice. A foundation of responsive feeding is that optimal eating behaviors that will be beneficial throughout life are learned in the formative years. High sugar consumption during these early years undermines optimal satiety programming through overriding satiety cues, resulting from increased sugar cravings that do not correspond to hunger (Fazzino and Kong 2023; Griebel-Thompson et al. 2023; Jansen et al. 2020). It has been found that children of parents who follow responsive eating practices during early infancy have a lower proportion of sugar consumption throughout life (Hübner and Bartelmeß 2024). Importantly, research has found links between sugar consumption and increased risk of obesity and other health issue—such as diabetes and metabolic disease—later in life (Hübner and Bartelmeß 2024; Kong et al. 2021; Magriplis et al. 2021; WHO 2023a). The Samoan guidelines, like most international guidelines, strongly advise against added sugar for infants under the age of 12 months. The CDC, U.S. Department of Agriculture (USDA), and U.S. Department of Health and Human Services all recommend that children under the age of 2 years avoid all added sugars (CDC 2024b; Dietary Guidelines Advisory Committee 2020). Even after the introduction, they recommend that children 2 years and older limit added sugars to less than 10% of their daily caloric intake (Dietary Guidelines Advisory Committee 2020). A study by Moore et al. (2019) have found that infants for which sugar is included in their diet have a higher weight-for-length z-score than their peers who did not consume any sugar. Additionally, a longitudinal study found that infants who regularly consumed added sugar in their first year of life were at a higher risk of obesity in later childhood (Jardí et al. 2019). As the intake of sugar during infancy is linked with an increase in excess adiposity in childhood and later obesity risk, the practice of using these foods as a tool to encourage behavior change (around food or otherwise) may be another important target for educational intervention.

There were differing views on when to introduce solids to an infant (see Figure 3) that ranged from 4-months up until 1 year. The age in which solids are introduced to an infant has implications for growth and development. That one-fifth of the participants stated that the ideal age in which to begin introducing solid foods to an infant is 4- or 5-months may have implications for responsive feeding counseling, satiety programming, and later body composition—if development milestones are not indicating that the infant is ready for early introduction of foods based on their development (Pérez-Escamilla et al. 2019). For example, studies have linked the introduction of complementary foods earlier than the recommended 6 months with an increase in obesity risk in later life (Mameli et al. 2016). At the opposite end of the scale, one-fifth of the participants stated that they believed the ideal age of weaning to be 1 year old. However, we believe that this result may not represent the views of the participants and instead may be a miscommunication of what is meant by the word “solids” in this context. Instead of meaning any food that is not human milk, it may have been misconstrued to be food that is hard or requires working the jaw.

Of interest in our findings, there was a change in responses regarding inclusion of infants with the family at mealtimes across the three age groups (< 6 months, 6–12 months, and 1–2 years) presented to participants (see Figure 2). While a higher proportion of participants stated they included infants under the age of 6 months at the family mealtimes than infants aged 6–12 months, the largest difference was seen in the attitudes toward inclusion in the family mealtimes at age 1–2 years. It would be interesting to explore further why there is a shift in attitudes after the first year of life and if this includes a change in the foods that infants are being introduced to during mealtimes. Promoting inclusion at family mealtimes is important as it has been shown that eating at the family table encourages the development of responsive feeding techniques and decreases the risk of obesity (Anderson and Whitaker 2010; Casazza et al. 2015; Hammons and Fiese 2011). This is believed to be due to the increased parental involvement that allows for monitoring appropriate intake and modeling healthy eating practices and diet quality (Casazza et al. 2015). In our sample, about half of the participants stated that they do not include infants aged 6–12 months in family mealtimes. As this is the period of time when infants are being introduced to solids for the first time, it is also an important period for responsive feeding and the modeling of optimal eating behaviors from parents. The lack of inclusion in mealtimes during this vital learning period may be hindering the development of responsive eating practices in this population. More research into how family mealtime inclusion interacts with responsive eating practices would be of interest.

How parents react toward their infant’s food preferences or to infant crying and/or fussiness has been shown to influence the success of implementing responsive feeding techniques. Our results found a mix of responsive and non-responsive feeding practices. While many of the participants discussed responses that were conducive to responsive feeding techniques—such as continuing to expose infants to food they previously rejected or using calming and/or distraction techniques in response to crying/fussiness, techniques that are counter to responsive feeding were equally discussed. Many participants discussed using “desirable” foods—often sugary- and unhealthy-foods—as bribes to either get their infants to eat what was currently offered to them or to control crying/fussiness. Research has found that using food as a reward or bribe is linked with decreased intake of fruits and vegetables in later life, an increased risk of overconsumption of sugar-rich foods, and a higher probability of increased food fussiness and emotional eating patterns in adolescence and adulthood (Hübner and Bartelmeß 2024; Jansen et al. 2020). Additionally, many participants mentioned pressuring their infants to eat and finish the plate or punishing signs of fussiness. Responsive feeding practices advise against this technique as it has been shown that coercive control of food intake leads to unhealthy eating behaviors in children that continues into later life as a result of undermining the infant’s development of self-regulatory skills (Hübner and Bartelmeß 2024; Savage et al. 2018; Vaughn et al. 2016). Overall, these non-responsive feeding practices discussed by the participants may contribute to suboptimal satiety development in the infant. We suggest these topics should be further explored in future research within this population.

Additionally, the theme around the use of technology that arose in the focus groups can be seen as hindering inclusion of infants at family mealtimes and was often cited as a tool to be used to distract the children so that the adults could eat in a calmer environment. Technology use has been shown to reduce the efficacy of responsive feeding practices, through hindering the ability of both the children and their parents to identify hunger and satiety cues in order to accurately respond to them (Ventura et al. 2023). Additionally, the American Academy of Pediatrics (AAP) maintains that the overuse of media—particularly television—and screentime in children and adolescents contributes to a higher risk of obesity (American Academy of Pediatrics 2011). Although the AAP acknowledges that the mechanisms are still unclear, unhealthy eating and increased snacking behavior are associated with eating while viewing media. Research has found that a higher exposure to screen time in childhood increases obesity risk in adolescence and adulthood (Anderson and Whitaker 2010; Casazza et al. 2015; Strasburger et al. 2009; Viner and Cole 2005). Children who tend to watch television or other media during mealtimes have been shown to consume less fruit and vegetables, more high-density foods, and reduce their attunement to responsive feeding leading to overeating (American Academy of Pediatrics 2011; Blass et al. 2006; Casazza et al. 2015). As a result, we would suggest targeting parents in Samoa with key messages around the importance of screen- and technology-free mealtimes to promote healthy eating patterns and, in turn, decrease the associated obesity risk.

## 5 | Limitations and Future Research Suggestions

While this study exhibits many strengths, there are a few limitations. One limitation is the potential confusion over the use of the word “solids” to mean any food that is not human milk, which may have caused a misrepresentation of weaning age preferences in our results. This should be explored further in work with the population to ensure that the correct definition of the word is being portrayed in interviews and questionnaires examining the weaning period in Samoa. Secondly, as discussed earlier, while the residence of Apia Urban Area is similar between the two samples (interview vs. focus group), the distribution of other census regions for these two samples differed, with lower residence of Northwest Upolu region (subsample; 40% vs. initial sample; 53%) and higher residence in the rest of Upolu (subsample; 40% vs. initial sample; 27%). While slight, this difference in socio-economic status that is associated with census region in Samoa may have influenced comparisons between the two samples. Lastly, while this research examined responsive feeding behaviors during the focus groups to assess what responsive feeding practices are being implemented by mothers in Samoa, only one participant specifically mentioned any awareness of implementing responsive feeding as such, despite prompting throughout the focus group questions. The one response came from a participant from the Apia urban area who was discussing her sixth child. It would be interesting to explore further if the participant’s location of residence or parity are factors in her understanding of responsive feeding.

To our knowledge, the health services in Samoa are not currently promoting responsive feeding practices. As many of the topics discussed by the participants were important aspects of responsive feeding practices, it is likely that while the concept is not well known in Samoa, participants are indeed practicing responsive feeding behaviors even if they do not refer to them as such. Further research is required to investigate if specific knowledge of responsive feeding practices is limited in Samoa or if the topic was not addressed appropriately in the questions that were asked. Additionally, as many reported learning infant feeding techniques from their family members—primarily their own mothers—it would be of interest to explore why some family teach responsive feeding practices and others do not. As responsive feeding can help regulate satiety and hunger responses from an early age and therefore potentially mitigate the risk for developing obesity in later life, it is an important area to explore in this population.

Despite the limitations, the results of this study can be used to develop suggestions for behavioral intervention strategies that support breastfeeding and weaning mothers to recognize infant hunger and satiety cues in order to promote healthy patterns of infant growth, as well as evidence-based messaging that fills gaps in public knowledge on human milk composition, responsive feeding practices (during breastfeeding and weaning), and the benefits for infants. To assist this, future research would benefit from interventions that introduce public health messages surrounding healthy foods for weaning, inclusion of infants at the family table, and reducing technology use at mealtimes, measuring any changes to responsive feeding outcomes as a result of disseminating this information.

## 6 | Conclusion

Our findings suggest that targeting families in Samoa to discuss the importance of including infants in family mealtimes, learning how to recognize and respond to hunger/satiety cues, and introducing new foods in a responsive way will help with promoting responsive feeding in this population. Many interventions for over- and under-weight in children focus on nutritional intervention (Black and Aboud 2011). However, early feeding practices may be a key area in which to address problems with eating behavior and satiety regulation before they have been established (Black et al. 2015). The information gained from the interviews and focus groups can be used to develop evidence-based messaging that strives to educate parents and caregivers in successful identification of hunger and satiety signals in infants, encourage the introduction of healthy and age-appropriate foods that complement current Samoan breastfeeding practices and childhood dietary patterns, and promote the inclusion of infants at family mealtimes in order to increase current responsive feeding practices. Findings can also be used to justify developing comprehensive responsive feeding guidelines and corresponding counseling materials in Samoa and neighboring nations as it has been done elsewhere (Pérez-Escamilla et al. 2017; UNICEF 2025).

## Supplementary Material

Supplementary

Additional supporting information can be found online in the Supporting Information section. **Data S1:** ajhb70241-sup-0001-Supinfo.docx.

## Figures and Tables

**TABLE 1 |:** Basic sample demographics.

	Individual interviews (*n* = 100)	Focus groups (*n* = 25)
Maternal and infant characteristics	Mean ± SD; range or percentage	Mean ± SD; range or percentage
Maternal age (years)	27.7 ± 6.3; Min: 18, Max: 42	27.1 ± 5.9; Min: 18, Max: 39
Infant age (weeks)	15.9 ± 4.5; Min: 6.5, Max: 23.5	—
Infant age (months)	2.7 ± 1.0; Min: 1, Max: 4	—
Female infants	62.7%	52%
Maternal parity (number of children)	3.4 ± 1.8; Min: 1, Max: 10	3.3 ± 1.6; Min: 1, Max: 7
*Marital status*		
Never married	22.5%	20%
Currently married	75.5%	80%
Cohabiting	1%	0%
Widowed	1%	0%
Divorced	0%	0%
*Highest education*		
No formal schooling	0%	0%
Less than primary school	2%	4%
Primary school completed	7%	4%
Secondary school/college completed	83%	80%
University completed	8%	12%
Postgraduate completed	0%	0%
*Maternal employment status*		
Employed	1%	—
Unemployed	99%	—
*Partner employment status*		
Employed	50%	—
Unemployed	49%	—
Not applicable	1%	—
*Census region*		
Apia Urban Area	19%	20%
Northwest Upolu	53%	40%
Rest of Upolu	27%	40%
Not reported	1%	0%

**TABLE 2 |:** Infant feeding data.

Infant feeding data	Percentage
*Infant feeding routine*	
On demand	45%
Generally kept to set times	25%
Depends on the circumstances	30%
*Average length of breastfeeding bout*	
Less than 10 min	16%
10–19 min	27%
20–29 min	14%
30–39 min	41%
40–49 min	2%
50 or more minutes	0%
*“Do you feel confident that you could recognize if your baby is getting enough milk?”*	
Yes	88%
No	12%
*“Did anyone explain to you how to recognize that your baby is getting enough milk?”*	
Yes	39%
No	61%
*“Who explained this to you?”* *	
Own mother	38%
Doctor	26%
Own mother and doctor	33%
Friends	3%

* *n* = 39 responses.

## Data Availability

The data that support the findings of this study are available from the corresponding author upon reasonable request.
