# Supplementary material for: Nurturing Bonds: A Qualitative Exploration of Breastfeeding and Responsive Feeding Practices in Samoan Mother-Infants Dyads
Source: Am J Hum Biol. Author manuscript; Available in PMC 2026 May 15. (PMC13178251; doi:10.1002/ajhb.70241)
Supplement: Supplementary [file NIHMS2166816-supplement-Supplementary.docx]

**Supplementary Materials**

Supplemental 1: Interview Questions

**Maternal Interview Guide**

The objective of the interview is to collect Samoan mother’s knowledge and opinions on breastfeeding and infant satiety. Specifically, we are interested in how they assess their opinions on breastfeeding quality, how they gauge infant cues of hunger or fullness, and where this knowledge has been gained. Mothers will be interviewed during the home visit portion of data collection.

*Questions for Aim A (Breastmilk Quality and Concerns)*

- What do you think is in breastmilk?
- What do you believe you are passing to your child when you breastfeed them?
- What concerns do you have, if any, about what is being passed through breastmilk?

→ Have you always had those concerns?

*Questions for Aim B (Infant Satiety and Hunger Response)*

- How do you know when your baby is hungry?
  → What cues do your infant give?

→ Where and when did you learn these signs and signals?

- How do you know when your baby is full?
  → What cues do your infant give?

→ Where and when did you learn these signs and signals?

Supplemental 2: Focus Group Agenda

**Responsive Feeding Knowledge, Attitudes and Practices Among Samoan Mothers**

**Focus Group Discussion Guide**

Mothers will be selected if they are multiparous with a current child aged roughly 12 months. Women will be asked to join the focus group after completion of the home and laboratory visit portion of the study. The objective of the focus groups is to collect Samoan mother’s knowledge and opinions on breastfeeding, responsive feeding, and weaning practices. Specifically, we are interested in how they assess infant cues of hunger or fullness, how they decide what to feed their infants, and where this knowledge has been gained.

**Agenda for Focus Group**

**Section A**

**Intent:** The goal of section A is to gain an understanding of breastfeeding knowledge, responsive feeding knowledge of babies younger than 6 months, and weaning timing in the Samoan mothers.

1. What do you know about what babies younger than 6 months of age should eat?

a. Can you tell me what you know about breastfeeding?

i. What has made breastfeeding challenging for you?

ii. What has made breastfeeding easy for you?

2. How did you learn what babies younger than 6 months should eat?

a. What are other things that have helped you understand what they should eat?

3. How do you know when your baby younger than 6 months is hungry?

a. How do any of these cues change as babies get older?

b. How did you learn how to understand when your baby is hungry?

4. How do you know when your baby younger than 6 months is full?

a. How did you learn how to understand when your baby or young child is full?

b. What do babies 6 months – 1 year do when they are full?

5. At what age do children need to be introduced to solid foods?

a. How can you tell when a baby is ready to be introduced to solid foods?

**Section B**

**Intent:** The goal of section B is to gain information on maternal responsive feeding knowledge regarding infants aged between 6 months and 1 year and to explore experiences of introducing new foods to young children during weaning.

6. What do you know about what babies and young children between 6 months and 1 year
 should eat?

7. How did you learn what babies and young children of this age (6 months to 1 year) should
 eat?

a. What are other things that have helped you understand what they should eat?

8. How are children introduced to new foods in Samoa?

a. How can you tell if your child *does not* like the new food?

b. What do you do if your child *does not* eat his/her new food?

c. What do you do when your child *does* eat his/her new food?

d. What can you do to help your children learn to eat new foods?

e. How did you learn how to help your young child learn to eat new foods that they didn’t
 readily accept the first time offered?

f. Which new foods are easier for children to accept?

g. Which new foods are more difficult for children to accept?

**Section C**

**Intent:** The goal of section C is to gain information on mealtimes in Samoan families and the engagement of infants at different ages in family mealtime.

9. What is mealtime like for Samoan families? *(probe for if there are times when they eat in front
 of the TV or having distractions while eating like reading or using electronics)*

a. Are babies younger than 6 months included in family mealtime? If so, how?

b. Are babies between 6 months and 1 year included in family mealtime? If so, how?

c. Are young children between 1 and 2 years included in family mealtime? If so, how?

**Section D**

**Intent:** The goal of section D is to gain information on maternal strategies for responding to crying and/or fussiness in their infant across different ages.

10. How do you respond when your babies are crying or fussy?

a. How do you respond if babies who are 6 months to 1 year are crying or fussy?

b. How do you respond if they are young children ages 1 to 2 years and are crying or
 throwing a tantrum?

c. Could you tell me about your experience responding to young children crying or
 throwing a tantrum?

Supplemental 3: Focus Group Theme Coding Breakdown

**Human Milk Attitudes**

| **Section A** | | |
| --- | --- | --- |
| 1. **Food for babies less than 6 months** | | |
| Breastfeeding | Biological Components | |
|  | Best for baby | |
|  | Benefits | Protection |
|  |  | What mom eats |
|  |  | Healthy |
|  |  | Strong / Strength |
|  | Challenges | What mom eats |
|  |  | Breast pain |
|  |  | Baby sick |
|  |  | Biting |
|  |  | Return to work |

**Weaning Age**

| **Section A** | |
| --- | --- |
| 1. **Food for babies less than 6 months** | |
| Solids | Timing |
| 1. **How did you learn knowledge for under 6 months** | |
| Own parents / family | Mother |
|  | Parents |
|  | Grandmother |
|  | Sibling/s |
|  | Family members (does not define) |
| Hospital | |
| MOH | |
| TV | |
| Vaccination card | |
| Other | |
| 1. **Age for solids** | |
| Age example | |
| How to know | Teeth |
|  | Interest in food |
|  | Chewing |
| **Section B** | |
| 1. **How to learn what to feed 6-12 months** | |
| Own parents | |
| Vaccination card | |
| MOH | |
| TV | |
| Other | |

**Weaning Foods**

| **Section A** | |
| --- | --- |
| 1. **Food for babies less than 6 months** | |
| Solids | Types of food |
| **Section B** | |
| 1. **Food for babies** | |
| Soups / Soft / Liquid | |
| Sweet foods / Sugar | |
| Healthy | |
| Hold in hand | |
| Specific examples | |

**Introduction of New Foods**

| **Section B** | |
| --- | --- |
| 1. **Introduction to new foods** | |
| How to tell does not like | Waste / Refuse |
|  | Eats other food |
|  | Spit out |
|  | Facial expression |
| What to do if does not eat | Give different food |
|  | Offer a different way |
|  | Nothing |
| What to do if does eat | Try different way |
|  | Add other foods |
|  | Make available |
| How to help new foods | Familiarize / Keep trying |
|  | Incentive / Bribery |
|  | Punishment |
|  | Don’t force |
|  | Force |
|  | Encourage |
|  | Try new food / Variety |
| What new foods are easier | Fruit |
|  | Porridge |
|  | Soup |
|  | Sweets / Treats |
|  | Vegetables |
| What new foods are harder | Dry |
|  | Plain |

**Mealtimes**

| **Section C** | |
| --- | --- |
| 1. **Mealtimes** | |
| Babies under 6 months | Yes |
|  | No |
|  | How |
| Babies 6 – 12 months | Yes |
|  | No |
|  | How |
| Children 1 – 2 years | Yes |
|  | No |
|  | How |
| Technology use | Distraction |
|  | Calming / Ease |

**Crying/Fussiness Response**

| **Section D** | |
| --- | --- |
| 1. **Response when crying or fussing** | |
| 6 – 12 months | Comfort |
|  | Breastfeed |
|  | Distract |
|  | Find problem |
|  | Respond |
| 1 – 2 years | Calm / Comfort |
|  | Find problem |
|  | Technology / Phone / iPad / TV |
|  | Punishment |

**Sweet Food / Candy**

| **Section A** | |
| --- | --- |
| 1. **Food for babies less than 6 months** | |
| Solids | Types of food |
| **Section B** | |
| 1. **Food for babies** | |
| Sweet foods / Sugar | |
| Specific examples | |
| 1. **Introduction to new foods** | |
| How to help new foods | Incentive / Bribery |
| What new foods are easier | Sweets / Treats |
